# Supplementary material for: Cancer cells surviving cisplatin chemotherapy increase stress-induced OMA1 activity and mitochondrial fragmentation
Source: Sci Rep. 2026 Jan 6;16:3636. doi: 10.1038/s41598-025-33677-1 (PMC12848309; doi:10.1038/s41598-025-33677-1)

Supplementary Information

Cancer cells surviving cisplatin chemotherapy increased stress-induced OMA1 activity and mitochondrial fragmentation

Melvin Li[1,2*], Chenille A. McCullum[1], Louis T.A. Rolle[1], Qin Ni[3,4], Zhuoxu Ge[3,4], Sean X. Sun[3,4], Kenneth J. Pienta[1,2], Sarah R. Amend[1,2]

[1]Cancer Ecology Center, The James Brady Urological Institute, Johns Hopkins School of Medicine, Baltimore, MD 21287
[2]Pharmacology and Molecular Sciences Program, Johns Hopkins School of Medicine, Baltimore, MD 21287
[3]Institute for NanoBio Technology, Johns Hopkins University
[4]Department of Mechanical Engineering, Johns Hopkins University
*Correspondence: Melvin Li (mli154@jh.edu)

Author Contact Information
Melvin Li (mli154@jh.edu)
Chenille A. McCullum (cmccul17@jh.edu)
Louis T.A. Rolle (lrolle1@jh.edu)
Qin Ni (qni4@jhu.edu)
Zhuoxu Ge (zge6@jhu.edu)
Sean X. Sun (ssun@jhu.edu)
Kenneth J. Pienta (kpienta1@jhmi.edu)
Sarah R. Amend (samend2@jhmi.edu)

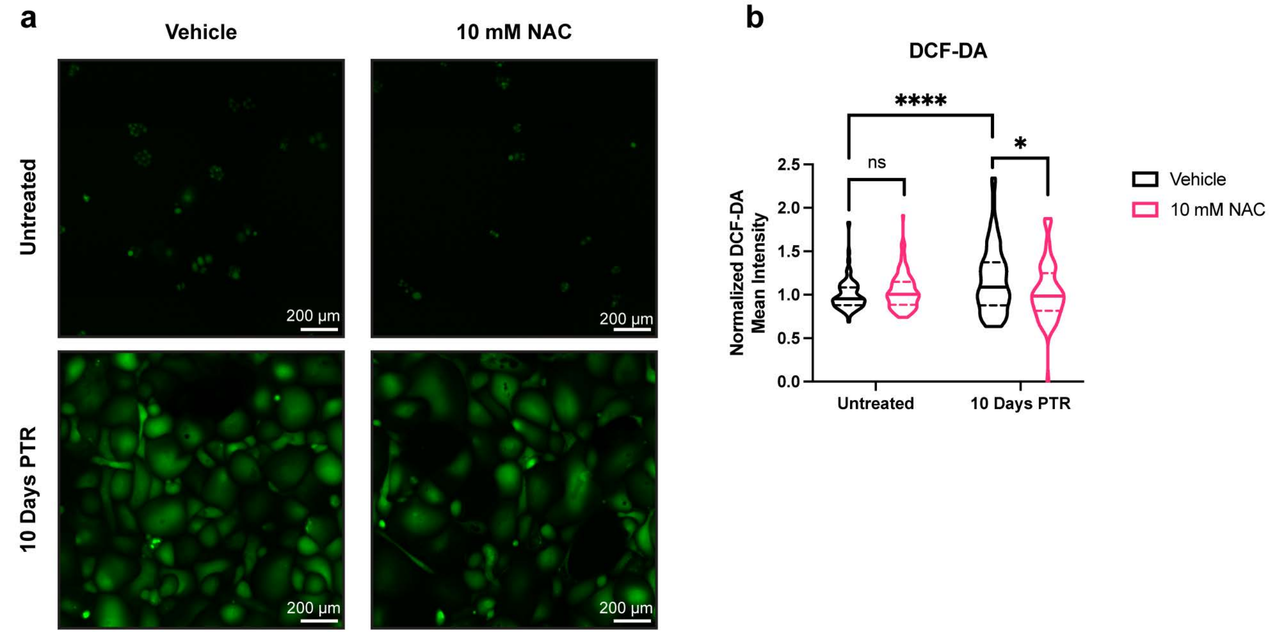

Supplementary Fig. S1. N-acetyl cysteine decreases levels of reactive oxygen species in cells 10 Days Post-Treatment Removal. (**a**) Representative DCF-DA fluorescence images of cells treated with N-acetyl cysteine and vehicle. (**b**) Quantification of mean fluorescence intensity of DCF-DA between indicated groups.

Supplementary Fig. S2. Glycolysis and hypoxia gene sets are enriched in cells 10 Days PTR. Gene set enrichment analyses from single-cell RNA sequencing data of cells 10 Days PTR and untreated cells. (**a**) Enrichment plot of the HALLMARK GLYCOLYSIS gene set. (**b**) Enrichment plot of the HALLMARK HYPOXIA gene set.

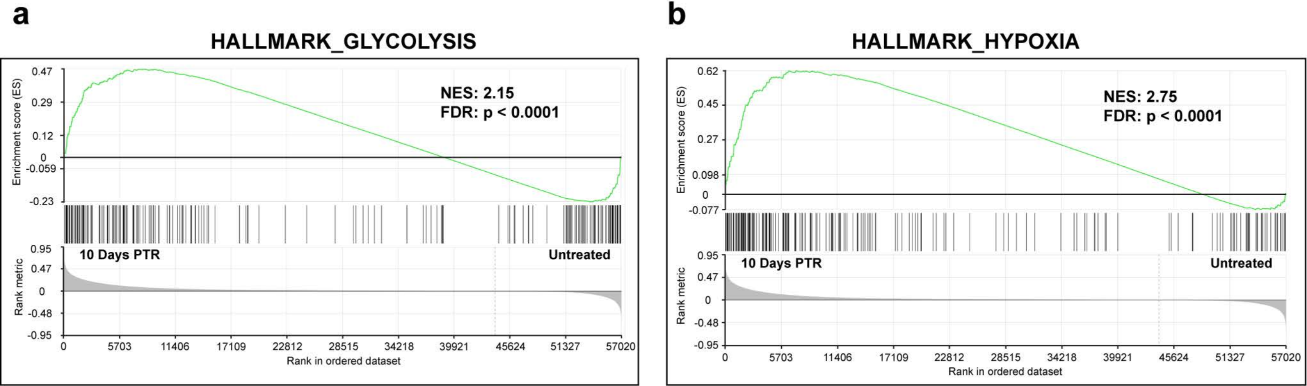

Supplementary Fig. S3. Uncropped blot images from Figure 2a. Boxed areas delineate the cropped portion displayed in Figure 2a.

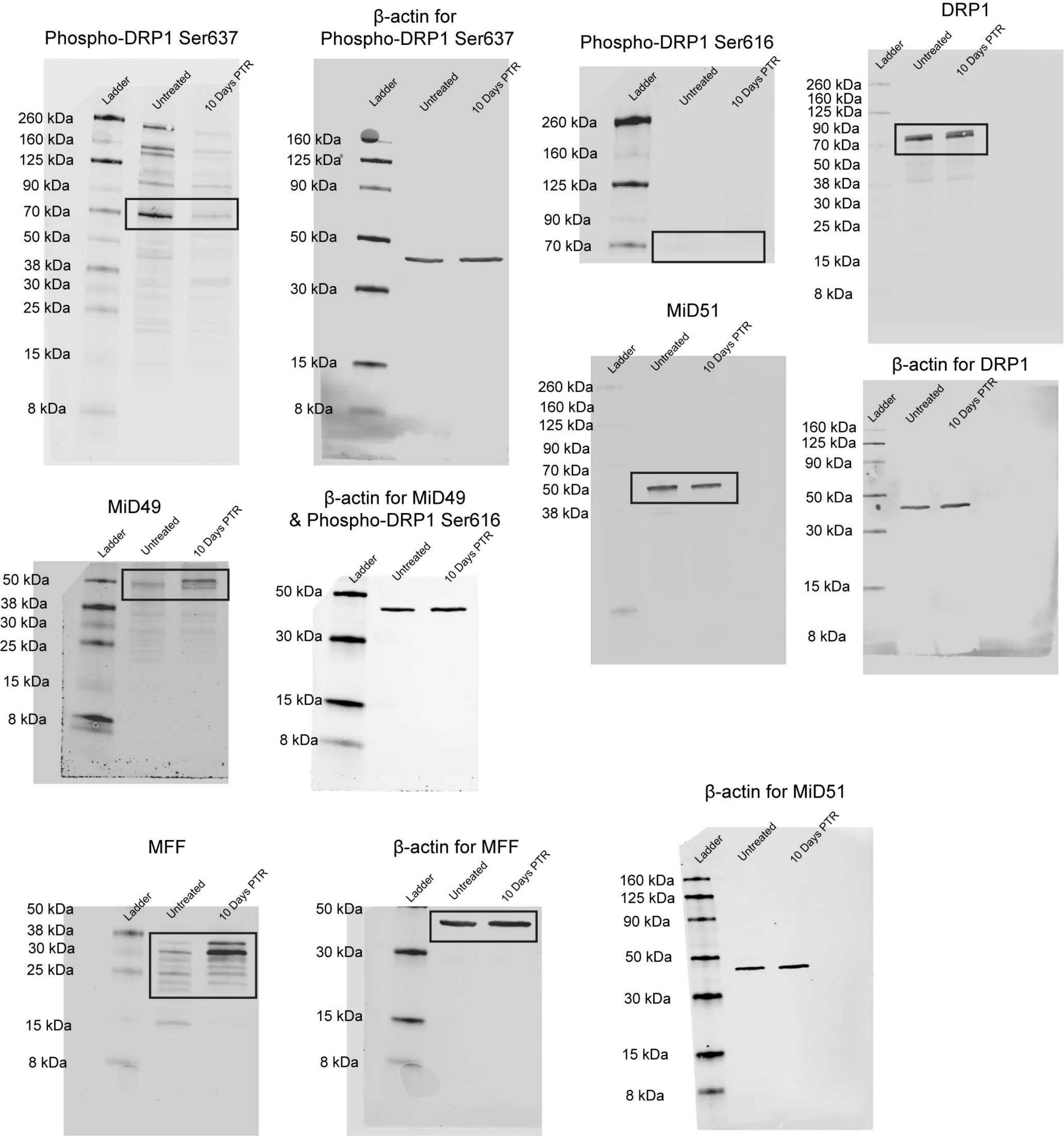

Supplementary Fig. S4. Uncropped blot images from Figure 3a. Boxed areas delineate the cropped portion displayed in Figure 3a.

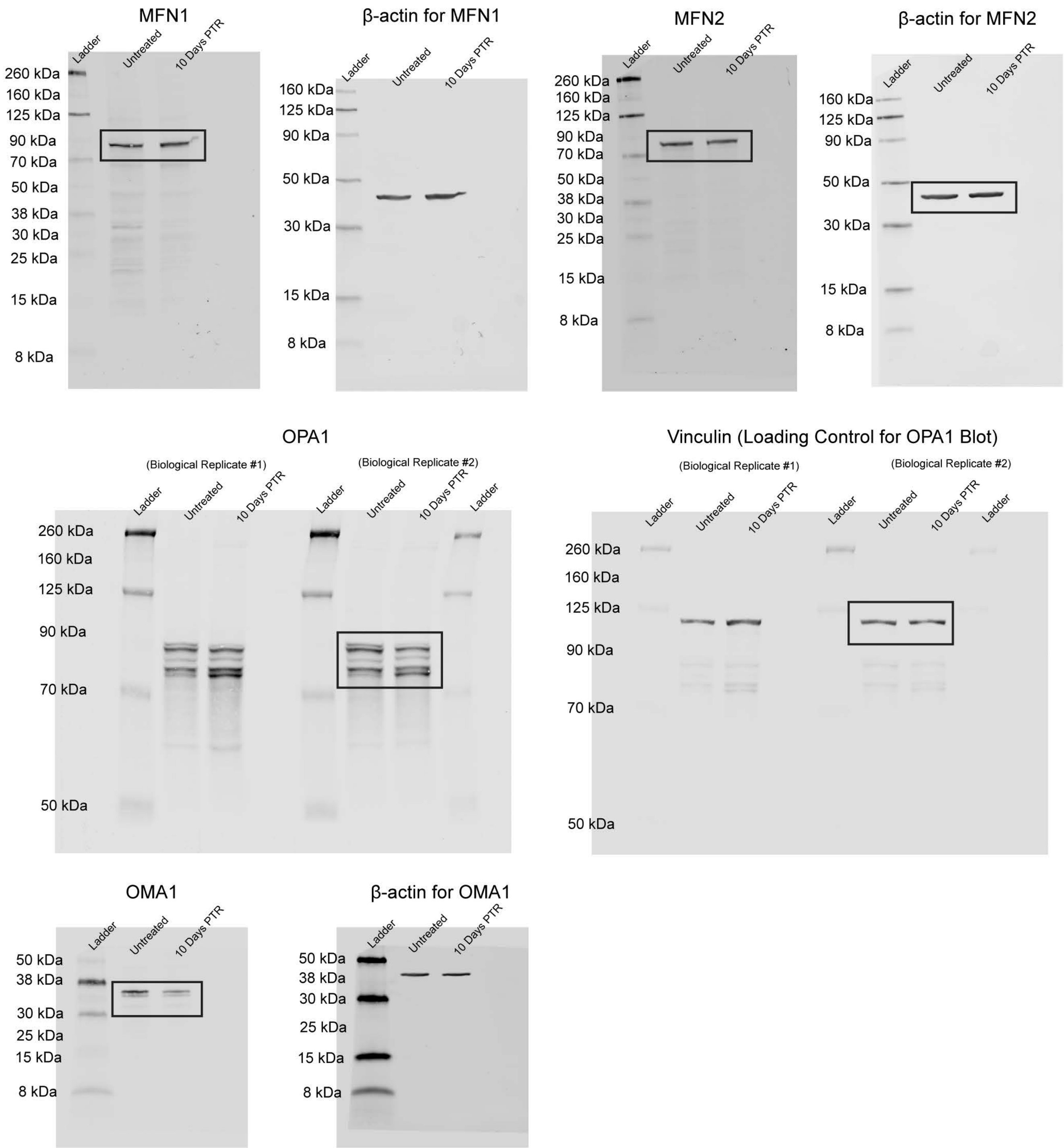

Supplementary Fig. S5. Uncropped blot images from Figure 5a. Boxed areas delineate the cropped portion displayed in Figure 5a.

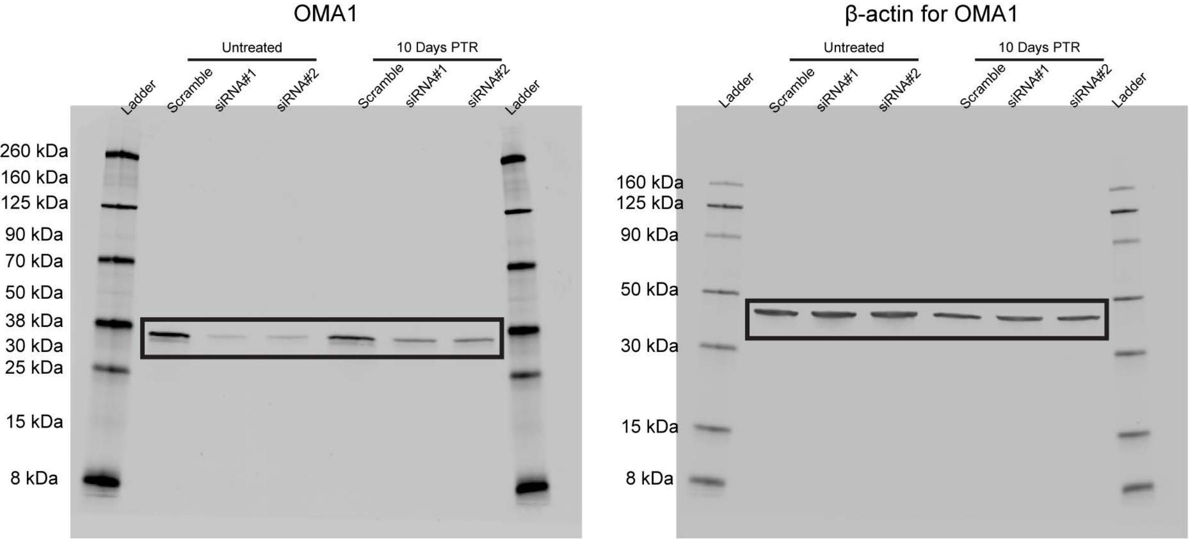

Supplement: Supplementary file 1 — Supplementary Information 1. [file 41598_2025_33677_MOESM1_ESM.pdf]
